# Frequency, associated factors and outcome of multi-drug-resistant intensive care unit-acquired pneumonia among patients colonized with extended-spectrum β-lactamase-producing Enterobacteriaceae

**DOI:** 10.1186/s13613-017-0283-4

**Published:** 2017-06-12

**Authors:** Keyvan Razazi, Armand Mekontso Dessap, Guillaume Carteaux, Chloé Jansen, Jean-Winoc Decousser, Nicolas de Prost, Christian Brun-Buisson

**Affiliations:** 1 Assistance Publique-Hôpitaux de Paris, CHU Henri Mondor, DHU A-TVB, Service de Réanimation Médicale, 94010 Créteil, France; 2 Université Paris Est Creteil, IMRB, GRC CARMAS, 94010 Créteil, France; 3 Assistance Publique-Hôpitaux de Paris, CHU Henri Mondor, Contrôle, Epidémiologie et Prévention de l’Infection, CEPI, 94010 Créteil, France; 4 Assistance Publique-Hôpitaux de Paris, CHU Henri Mondor, Département de Virologie, Bactériologie, Parasitologie-Mycologie, 94010 Créteil, France

**Keywords:** ESBL infection, Nosocomial pneumonia, Risk factors, ESBL colonization

## Abstract

**Background:**

We assessed prevalence, associated factors and prognosis of extended-spectrum beta-lactamase-producing Enterobacteriaceae pneumonia acquired in intensive care unit (ESBL-PE pneumonia) among carriers. Variables associated with nosocomial pneumonia caused by carbapenem-resistant bacteria (CRB) were also assessed.

**Methods:**

A 6-year prospective study (May 2009–March 2015) in the medical ICU of an 850-bed university-affiliated hospital was conducted.

**Results:**

Of the 6303 patients admitted, 843 (13.4%) had ESBL-PE carriage detected. Among carriers, 111 (13%) patients developed ICU-acquired pneumonia of whom 48 (43%) had ESBL-PE pneumonia (6% of carriers). By multivariable analysis, SAPS II at admission >43 [OR 2.81 (1.16–6.79)] and colonization with *Enterobacter* sp. or *K. pneumoniae* species [OR 10.96 (2.93–41.0)] were independent predictive factors for ESBL-PE pneumonia in colonized patients, whereas receipt of >2 days of amoxicillin/clavulanic acid during the ICU stay [OR 0.24 (0.08–0.71)] was protective. Patients with ESBL-PE pneumonia had a higher SOFA score (*p* = 0.037) and more frequent septic shock at pneumonia onset (*p* = 0.047). However, ESBL-PE pneumonia was not an independent predictor of mortality. Twenty-five patients had pneumonia caused by CRB. Chronic renal insufficiency, administration of third-generation cephalosporin within the past 3 months, acute respiratory distress syndrome before pneumonia and prior therapy with a carbapenem or fluoroquinolones were associated with CRB pneumonia in this selected population.

**Conclusions:**

Although few ESBL-PE carriers developed ESBL-PE pneumonia overall, a high proportion of pneumonia were caused by ESBL-PE in carriers developing ICUAP. ESBL-PE pneumonia was not an independent predictor of mortality. As pneumonia caused by CRB is increasing, knowledge of factors associated with ESBL-PE or CRB pneumonia may help empiric therapy of pneumonia among ESBL-PE carriers.

**Electronic supplementary material:**

The online version of this article (doi:10.1186/s13613-017-0283-4) contains supplementary material, which is available to authorized users.

## Background

In gram-negative pathogens, beta-lactamase production remains the most important contributing factor to antimicrobial resistance. Since the beginning of the century, the prevalence of infection with extended-spectrum β-lactamase-producing Enterobacteriaceae (ESBL-PE) dramatically increased [1]. Such infections have been often associated with severe adverse clinical outcomes, including increased mortality, prolonged hospital stay and increased costs [2, 3]. However, the impact of ESBL-PE infection on mortality of patients with ICU-acquired pneumonia (ICUAP) remains equivocal [4]. These adverse outcomes have been related, at least in part, to delayed effective therapy [5]. Consequently, carbapenems are increasingly used by intensivists as empiric therapy for hospital-acquired sepsis. This vicious circle of bacterial resistance already contributes to the worrying global dissemination of carbapenemase-producing Enterobacteriaceae, especially among *Klebsiella pneumonia* [6] and to the risk of colonization or infection with non-fermenting carbapenem-resistant bacteria [7, 8]. New agents against these multi-drug-resistant bacteria are scarce, and the intensivist’s armamentarium is close to a dead-end without the cautious use of carbapenems [9].

In many ICUs, screening for ESBL-PE carriers is routinely performed, essentially for the implementation of isolation precautions. Therefore, intensivists often have the knowledge of ESBL-PE carriers, which incite them to cover these organisms empirically when ICUAP is clinically suspected [10]. Universal coverage of ESBL-PE in carriers developing pneumonia may, however, lead to overusing carbapenems (because all ICUAP in ESBL-PE carriers are not due to ESBL-PE), thus fostering selection of carbapenem-resistant bacteria [8]. A better knowledge of the prevalence and associated factors for ESBL-PE related ICUAP would help physicians to refine their therapeutic approach. The primary aim of our study was to determine, among ESBL-PE carriers, the prevalence, associated factors and clinical impact of ESBL-PE pneumonia. The secondary aim was to determine factors associated with ICUAP caused by carbapenem-resistant bacteria (CRB).

## Methods

### Setting and patients

This 6-year prospective study (May 2009–Mar 2015) was conducted in the medical intensive care unit of an 850-bed university hospital. Screening for ESBL-PE carriage is routinely performed on ICU admission and during the hospital stay, but no specific isolation precautions are used for patients with ESBL-PE recovered from screening cultures [11].

All patients having ESBL-PE carriage on admission or acquired during the ICU stay and developing pneumonia were included in this study. This observational study was approved by the Ethics Review Board of the French society for respiratory medicine (Société de Pneumologie de Langue Française), and informed consent was waived.

Rectal swabs were collected from each patient within 24 h of ICU admission and then twice weekly up to June 2011 and weekly thereafter for the duration of hospitalization in the ICU. Rectal swab samples were screened for ESBL-PE on chromogenic agar (Oxoid Ltd, Cambridge, UK; Biomérieux, Courtaboeuf, France), and ESBL production was confirmed by the double-disc synergy method using ceftazidime/cefotaxime and clavulanic acid. As a result, attending intensivists knew the species and susceptibility profile of ESBL-PE colonizers but not their MICs (especially for piperacillin-tazobactam). Data on all patients with ESBL-PE colonization or infection were prospectively collected.

### Demographic, clinical and laboratory data

A detailed clinical profile of each patient was established. The following data were collected: demographic characteristics, which included sex, age, simplified acute physiology score (SAPS II) [12], location before ICU admission, main reason for admission, hospitalization and administration of antibiotics in the previous year (stratified according to receipt within 3 months of admission or earlier), antibiotic class received and duration of antibiotic exposure, surgery in the previous year, presence of underlying diseases and Charlson comorbidity index [13], and presence of indwelling devices for more than 24 h before ICU admission. Results of cultures of respiratory tract secretions samples taken for diagnosis of pneumonia and susceptibility profile of microorganisms recovered were recorded.

### Intensive care unit-acquired pneumonia

ICUAP was clinically suspected on the following usual criteria occurring 48 h or more after admission: new or worsening infiltrates on the chest roentgenogram, systemic signs of infection, purulent secretions, and impaired oxygenation. ICUAP was confirmed from cultures of respiratory tract secretions sampling of distal airways obtained before administering new antibiotics, using a protected telescoping catheter or bronchoscopy, both with quantitative cultures (10^3^ and 10^4^ colony-forming units/mL for protected telescoping catheter and broncho-alveolar lavage, respectively). Ventilator-associated pneumonia (VAP) was defined as an ICUAP arising more than 48 h after mechanical ventilation initiation.

Patients having colonization detected only after developing ESBL-PE-related pneumonia were excluded. If a patient with known ESBL colonization developed more than one ICUAP, only the first ESBL-PE pneumonia episode was analysed (the last episode was considered if all ICUAP episodes were non-ESBL-PE related). First-line antibiotic delivered within the first 24 h following ICUAP was deemed appropriate if the isolated pathogen was susceptible to at least one drug administered (including aminoglycosides alone). During the study period, our strategy for management of VAP/HAP was derived from that recommended by the ATS-IDSA Guidelines for the management of adults with hospital-acquired, ventilator-associated and healthcare-associated pneumonia [14]. Combination therapy was not systematic. Since the incidence of MRSA was low (<5%), routine empiric antibiotic against MRSA was avoided. De-escalation was systematically performed if possible to limit carbapenem use.

The clinical impact of ESBL-PE pneumonia was assessed from the rate of inappropriate empiric therapy, the severity of the host response, mortality rate and length of stay.

### Statistical analysis

Results are reported as median and interquartile range (25th–75th percentiles) or numbers with percentages. Univariable analysis first assessed the association between each variable and ESBL-PE ICUAP. Initial bivariate statistical comparisons were conducted using the Chi-square or Fisher’s exact test for categorical data and the Mann–Whitney *U* test for continuous data. To identify patients’ characteristics associated with ESBL-PE pneumonia, we used multivariable logistic regression with a backward procedure. Non-redundant variables selected by bivariate analysis (*p* < 0.10) and considered clinically relevant were entered into a logistic regression model. Considering the number of events, a maximum of 6 variables was entered in a two-step model, first including baseline characteristics and characteristics of colonization/infection, then antibiotic exposures. Results are expressed as crude and adjusted odds ratios (OR) with their 95% confidence intervals (CI). A *p* value <0.05 was considered statistically significant. A sensitivity analysis restricted to ICUAP occurring only after known colonization was performed, thus excluding patients with pneumonia and ESBL-PE colonization diagnosed on the same day.

A second analysis assessed the association of each variable with CRB-related ICUAP. Finally, univariable Cox proportional hazards regression was used to evaluate predictors of sixty-day mortality. Results were reported as hazard ratios and 95% confidence intervals. Statistical analyses were performed with the use of Stata software, version 13.1 (StataCorp, College Station, TX, USA).

## Results

A total of 6303 patients were admitted to our medical ICU during the study period. Of these, 597 had ESBL-PE carriage detected on the admission screening sample (9.5%), mostly with *E. coli* alone (*n* = 340, 57%); 246 acquired ESBL-PE carriage, while in the ICU (246/5706 = 4.3%) mostly with *E. cloacae* or *K. pneumonia* (*n* = 192, 78%). ESBL-PE incidence density carriage was about 20 per 1000 hospital days and did not vary substantially along the study period. The incidence density rate of ICU-acquired ESBL colonization was 5.8 per 1000 hospital days. Among the colonized patients, 483 (81%) were mechanically ventilated.

Nine patients were excluded because they developed ESBL-PE pneumonia before detection of ESBL-PE carriage; all occurred during the period with weekly screening policy. One hundred and eleven patients had 157 episodes of ICUAP diagnosed at the same time or after carriage, including 54 due to ESBL-PE. Forty-eight patients (43%) had ESBL-PE-related ICUAP (39 during the first episode after colonization and 9 during a later episode of pneumonia); the remaining 63 (57%) patients had pneumonia caused by another microorganism. The first ESBL-PE-related ICUAP and the last episode of ICUAP in patients without ESBL-PE ICUAP were included in the analysis, including 98 VAP (88%) and 13 (12%) pneumonia in non-mechanically ventilated patients. All but one patient with ESBL-PE pneumonia (98%) had rectal carriage with the same species. ESBL-PE pneumonia occurred in 23 of the 597 (3.8%) carriers detected at admission and in 25 of the 246 (10%) patients with acquired ESBL-PE carriage. The incidence of ESBL-PE ICUAP for patients colonized with *E.cloacae/K. pneumonia* was 10‰ days at risk (between colonization and discharge) in the ICU, higher than in patients colonized with *E. coli* alone (1‰). Infection occurred after a median length of stay in ICU of 12 days [7–15], and a median of 6 days [3–11] after the detection of ESBL-PE carriage.

### Factors associated with ESBL-PE pneumonia among patients with colonization

 Additional file 1: Table S1 shows the main characteristics of patients and variables associated with ESBL-PE pneumonia among carriers. By multivariable analysis (Table 1), SAPS II at admission >43 and colonization with *Enterobacter* or *K. pneumoniae* species were independent predictive factors for ESBL-PE pneumonia in patients with colonization, whereas receipt of >2 days of amoxicillin/clavulanic acid during the ICU stay was protective (thus predictive of another microorganism than ESBL-PE). Amoxicillin/clavulanic acid was started 13 [8–23] days before ICUAP (Additional file 1: Table S1).

**Table 1:** Multivariable analysis of factors associated with ESBL-PE pneumonia among 111 patients with ESBL-PE colonization

Associated factors	AOR	95% CI	*p*
SAPS2 > 43	2.81	1.16–6.79	0.022
>2 days amoxicillin/clavulanic acid in ICU	0.24	0.08–0.71	0.010
Colonization with *E.cloacae* or *K. pneumoniae*	10.96	2.93–41.0	<0.0001

The multivariable model showed a good calibration as assessed by the Hosmer and Lemeshow goodness-of-fit test (*χ* ^2^ = 4.9, *p* = 0.30) and a fair discrimination as assessed by the receiver operating characteristics curve (area under the curve = 0.80)

Results did not differ in the sensitivity analysis excluding the six patients in whom ICUAP occurred on the same day as the detection of ESBL-PE colonization (see Additional file 1: Table S2).

### Treatment of ICUAP among patients with prior colonization

Forty-eight ESBL-PE carriers had ESBL-PE pneumonia, mostly caused by *Enterobacter* (27%) or *K. pneumoniae* (25%), alone or with other bacteria (39%), essentially non-fermenting gram-negative bacilli (Additional file 1: Table S3). ESBL-producing *E. coli* pneumonia was uncommon. Six patients had pneumonia with both CRB and ESBL-PE. First-line therapy with carbapenem was prescribed for 36 patients (75%) with ESBL-PE pneumonia and only 23 (37%) patients without ESBL-PE pneumonia. Appropriate therapy was given to most patients without difference between ESBL-PE infections, ESBL-PE without non-fermenting gram-negative bacilli (NF-GNB) infections and the remainders (77, 84 and 76%, respectively). Twenty-five (23%) of the 111 patients had CRB pneumonia, mostly (92%) due to NF-GNB. First-line antimicrobial therapy was less often appropriate for patients who developed CRB pneumonia as compared to others (54 vs. 83%, *p* = 0.014).

### Risk factors for CRB pneumonia

Bivariate analyses of variables associated with CRB pneumonia are shown in Additional file 1: Table S4. Chronic renal insufficiency, administration of third-generation cephalosporin within the past 3 months, ARDS before pneumonia and prior therapy with a carbapenem or fluoroquinolones during the ICU stay were associated with CRB pneumonia in patients with ESBL-PE colonization.

### Outcome

**Fig. 1:**
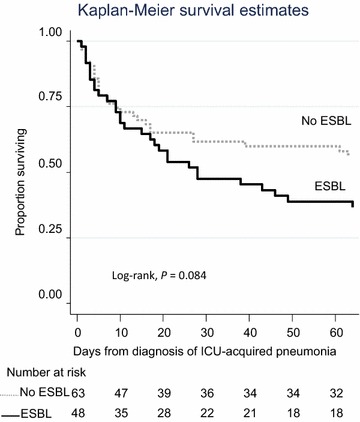
Sixty-day survival in patients with ESBL carriage and ICU-acquired pneumonia

Patients with ESBL-PE pneumonia had a higher SOFA score, more often septic shock at pneumonia onset and higher in-ICU mortality (58 vs. 38%; *p* = 0.034) (Table 2). However, the log-rank test failed to show a difference in mortality at 60 days after pneumonia between the two groups (*p* = 0.08) (Fig. 1). Furthermore, ESBL-PE infection was not a predictive factor of 60-day mortality (univariable and multivariable Cox regression analyses, Table 3).

**Table 2:** Outcome associated with nosocomial pneumonia, according to aetiology (*n* = 111)

Variables	ESBL− (*n* = 63)	ESBL+ (*n* = 48)	*p* value
Septic shock	21 (33%)	25 (52%)	0.047
SOFA at ICUAP onset	4 [2–9]	7 [4–10]	0.037
Bacteraemia	5 (8%)	7 (15%)	0.26
Appropriate initial first-line antimicrobial therapy^a^	48 (76%)	37 (77%)	0.91
Appropriate 1st beta-lactam	46 (73%)	31(65%)	0.34
Resolution of infection^b^	49 (78%)	35 (73%)	0.31
LOS in ICU, all patients	25 [18–41]	33 [19–60]	0.09
LOS in ICU, survivors only	25 [22–41]	40 [27–80]	0.017
LOS in hospital, all patients	41 [23–70]	42 (20–84)	0.81
LOS in hospital, survivors only	57 [40–75]	62 [46–121]	0.29
Death in ICU	24 (38%)	28 (58%)	0.034
Death in hospital	27 (43%)	32 (67%)	0.013

*LOS* length of stay

^a^First-line antibiotic administered within the first 24 h following ICUAP was deemed appropriate if the isolated pathogen was susceptible to at least one drug administered (including aminoglycosides alone)

^b^Resolution of clinical signs and symptoms of pneumonia without documented microbiologic persistence and alive at day seven

**Table 3:** Cox regression (bivariable and multivariable) analyses of variables associated with death at sixty days

Variables	Bivariable analysis		Multivariable analysis	
	HR (95% CI)	*p* value	aHR (95% CI)	*p* value
SAPS2 > 43	1.76 (1.03–3.00)	0.038	1.93 (1.12–3.34)	0.018
Chronic pulmonary disease	1.68 (0.93–3.04)	0.086	–	
Liver cirrhosis	1.89 (0.86–4.17)	0.11	–	
Ab < 3 mo., broad-sp. > 10 d	2.21 (1.31–3.71)	0.003	–	
C3G < 3 mo	1.64 (0.93–2.90)	0.087	–	
Carbapenem < 3 mo	2.59 (1.11–6.06)	0.03	–	
Charlson > 2	1.75 (1.04–2.95)	0.034	–	
ESBL colonization at admission	1.56 (0.92–2.63)	0.10	–	
Septic shock associated with nosocomial pneumonia	2.86 (1.68–4.85)	0.0001	2.81 (1.66–4.78)	<0.0001
VAP	0.48 (0.24–0.96)	0.037	0.48 (0.24–0.98)	0.04
ESBL-PE ICUAP	1.57 (0.93–2.64)	0.091	1.15 (0.65–2.05)	0.64
ICU-acquired infection before ICUAP	0.51 (0.28–0.95)	0.033	0.52 (0.28–0.97)	0.04
Others antibiotics between colonization and pneumonia	1.49 (0.89–2.52)	0.13	–	
Appropriate empirical antimicrobial therapy^a^	1.05 (0.56–1.95)	0.88	0.66 (0.34–1.27)	0.22

*Ab* antibiotic, *broad-sp.* broad-spectrum, *3GC* third-generation cephalosporin; *iBL* beta-lactamase inhibitor, *mo* month, *VAP* ventilator-associated pneumonia, *ICUAP* ICU-acquired pneumonia, *<3 mo* within 3 months before ICU admission, *HR (95% CI)* hazard ratio interquartile range (25–75%)

^a^Antibiotic treatment was considered adequate if one or more antibiotics initiated for ICUAP were active against the causative microorganism on the basis of the antibiotic susceptibility profile of the strain

## Discussion

 The first finding from this 6-year study conducted in an era of highly endemic ESBL-PE rate is the high proportion (43%) of pneumonia caused by ESBL-PE in carriers having a first or later episode of ICUAP occurring after detection of ESBL-PE carriage. However, few ESBL-PE carriers developed ESBL-PE pneumonia altogether (48 after colonization and 9 before colonization among 843 carriers, 7%). Second, we found that patients having a SAPS II at admission >43 and colonization with ESBL-producing *Enterobacter* or *K. pneumoniae* species were at higher risk of developing ESBL-PE pneumonia, while having received a combination of aminopenicillin and beta-lactamase inhibitor favoured another aetiology, mostly non-fermenting gram-negative bacilli infection. This study is, to our knowledge, the first that identified factors for ESBL-PE- and CRB-associated ICUAP in patients with prior ESBL colonization based on prospectively collected and comprehensive information on patients’ characteristics as well as prior exposures to antibiotics and other risk factors prior to and during ICU admission. Our findings have implications for the empiric use of carbapenems for treating ICUAP, at a time when their use should be spared because of the increasing threat and associated risk of carbapenem resistance.

Only 7% of ESBL-PE carriers developed ESBL-PE pneumonia in our study, in keeping with previous studies [15]. By comparison, in the 15-month period between January 2014 and the end of the study (March 2015), 49 (4%) of 1192 patients without ESBL-PE colonization developed ICUAP. Eight (16%) were due to gram-positive bacteria, 20 (41%) were due to Enterobacteriaceae (no ESBL-PE), and 21 (43%) were due to NF-GNB or polymicrobial with NF-GNB, including 5 (24%) CRB and one ESBL-PE. However, in the current cohort, ESBL-PE infection was identified in about one-third (35%) of first pneumonia episodes occurring after detection of carriage. Our predictive factors for ESBL-PE pneumonia may help refine empiric therapy of ICUAP in ESBL-PE carriers. This is especially warranted when colonization is due to ESBL-producing *Enterobacter* or *K. pneumoniae,* species, which are frequently involved in hospital-acquired infections, may persist in the hospital environment and cause outbreaks [16]. Conversely, as previously shown, ESBL-producing *E. coli* is more rarely involved in ESBL-PE pneumonia (8%). The risk of ESBL-PE pneumonia in patients carrying ESBL-producing *E.coli* alone and not presenting with septic shock at pneumonia onset is low (6%). Indeed, previous studies showed that *K. pneumoniae* infections are associated with more serious illness than *E. coli* infections [17, 18]. In a recent study, ESBL-PE caused 17 VAP (40%) among patients with prior colonization [19]. The proportion of ICU-acquired ESBL-PE-positive respiratory samples was 34% among colonized patients [20]. In these studies, the negative predictive value (i.e., low risk of developing an ESBL-PE pneumonia in a patient without ESBL-PE colonization) was very high (>90%). Therefore, the systematic coverage of ESBL does not seem to be mandatory when treating a first episode of pneumonia in a non-colonized patient or colonized with ESBL-producing *E.coli* alone and not presenting with septic shock; in contrast, such coverage is needed in subsequent episodes, especially when the patient is a known ESBL carrier.

Intriguingly, the combination of aminopenicillin and beta-lactamase inhibitor was negatively associated with ESBL-PE pneumonia in carriers, indicating that this drug favoured another aetiology than ESBL-PE, mostly non-fermenting gram-negative bacilli. Indeed, antibiotics ineffective against *P. aeruginosa* significantly increase the risk of colonization or infection with *P. aeruginosa* [21, 22]. Most ESBL-PE were resistant or had intermediate susceptibility in vitro to aminopenicillin and beta-lactamase inhibitor. Thus, the apparent protective effect of aminopenicillin and beta-lactamase inhibitor on the occurrence of ESBL-PE pneumonia may not be attributed to antimicrobial efficacy, but rather to the selection of *P.aeruginosa* as a causative agent of pneumonia.

ESBL-PE carriage has been recently associated with higher carbapenem exposure than in non-carriers, even in the absence of infection [15]. Using carbapenem for all suspected pneumonia in ESBL-colonized patients may foster the emergence and spread of carbapenem-resistant bacteria [8, 23], which may in turn result in inappropriate therapy when secondary infection is caused by carbapenem-resistant non-fermenting gram-negative bacilli, as illustrated in our series in which most patients not having ESBL-PE infection essentially had *P. aeruginosa* infection, some of which were carbapenem resistant. Indeed, prior therapy with a carbapenem was confirmed in our population as associated with infection due to CRB [23]. Thus, the clinician is faced with the dilemma of covering the risk of ESBL-PE infection with carbapenem in patients having ESBL colonization, or risking inappropriate therapy if the causative microorganism proves to be a carbapenem-resistant GNB. Our identified factors associated with ESBL-PE or CRB pneumonia may help to choose empiric therapy, and combination antibiotic therapy should thus be considered in this situation, particularly after carbapenem exposure. Now in our unit, if an ESBL-PE carrier who previously received aminopenicillin and beta-lactamase inhibitor develops an ICUAP, we choose an antibiotic regimen active against *Pseudomonas aeruginosa* according to the severity and previous antimicrobial therapy received. Indeed, an antipseudomonal third-generation cephalosporin or ureidopenicillin is initiated for ICUAP without septic shock. A carbapenem is administered to patients presenting with ICUAP and septic shock, but combination therapy with an antipseudomonal third-generation cephalosporin and an aminoglycoside is preferred after previous carbapenem exposure.

ESBL-PE pneumonia was associated with higher SAPS II and more often shock at admission, as well as a higher SOFA score and more often septic shock at pneumonia onset. However, infection with ESBL-PE was not an independent predictor of mortality. Barbier et al. [15] recently found that infection with ESBL-PE was a predictor of fatal outcome in critically ill patients. However, the impact of ESBL resistance on mortality remains controversial and was not supported by other studies in ICUs [24, 25]. In a large European cohort in ICUs, antimicrobial resistance patterns of *Escherichia coli* had no additional effect on mortality [26]. A previous study showed that the severity of ICUAP did not depend on bacteria involved but seemed to be mainly related to patients’ clinical status (age and SOFA before ICUAP) [4]. Moreover, bacterial resistance did not affect VAP or ICUAP mortality [4, 27].

Our monocentric study certainly has a number of limitations. First, this study is monocentric, and thus its results may not be generalizable to other settings; however, the clinical profile and antibiotics consumption in our patients were similar to those of other French ICUs (Additional file 1: Tables S5, S6). Second, the screening policy changed during the study period, possibly explaining the lack of increasing ESBL-PE incidence density carriage over time. Molecular typing could have provided a better insight into the relation between colonization and infection. Finally, the relatively small number of ESBL-PE infections and our selected population may have limited our ability to identify an impact on mortality.

## Conclusions

 Although few ESBL-PE carriers developed ESBL-PE pneumonia overall, a high proportion of pneumonia were caused by ESBL-PE in carriers developing ICUAP. The study did not show a significant association between ESBL-PE pneumonia and 60-day mortality. Our analyses of risk factors for ESBL-PE and CRB pneumonia among ESBL-PE carriers may be useful for identifying which patients may warrant empiric therapy targeting these organisms.

## Additional file

**Additional file 1.**. Additional results (Tables S1 to S6).

## Abbreviations

ESBL-PE: extended-spectrum beta-lactamase-producing Enterobacteriaceae
CRB: carbapenem-resistant bacteria
ICUAP: ICU-acquired pneumonia
