# Supplementary material for: Frequency, associated factors and outcome of multi-drug-resistant intensive care unit-acquired pneumonia among patients colonized with extended-spectrum β-lactamase-producing Enterobacteriaceae
Source: Ann Intensive Care. 2017 Jun 12;7:61. doi: 10.1186/s13613-017-0283-4 (PMC5468364; doi:10.1186/s13613-017-0283-4)
Supplement: Supplementary file 1 — Additional file 1.. Additional results (Tables S1 to S6). [file 13613_2017_283_MOESM1_ESM.docx]

Additional File 1

**Frequency, associated factors and outcome of multi-drug resistant intensive-care acquired pneumonia among patients colonized with extended-spectrum β-lactamase producing Enterobacteriaceae**

Keyvan Razazi MD, Armand Mekontso-Dessap, MD, PhD, Guillaume Carteaux, MD, PhD, Chloé Jansen, PharmD, Jean-Winoc Decousser, PharmD, PhD, Nicolas de Prost MD, PhD, Christian Brun-Buisson, MD

This electronic supplement contains six Tables, detailing univariable analyses of variables associated with ESBL nosocomial, ESBL PE species, univariate analyses of variables associated with pneumonia due to a carbapenem-resistant bacteria, the sensitivity analysis excluding patients having ESBL-PE colonization and pneumonia identified on the same day, antibiotic consumption and Study Population Characteristics

**Table S1.** Univariable analyses of variables associated with ICU-acquired pneumonia due to ESBL-PE among patients with prior colonization. (n=111)

| **Variables** | **ESBL -**  **(n=63)** | **ESBL +**  **(n=48)** | **OR (95%CI)** | **P Value** |
| --- | --- | --- | --- | --- |
| Male gender | 47 (75%) | 34 (71%) |  | 0.66 |
| Age, median [IQR] | 61 [50-74] | 64 [50-74] |  | 0.82 |
| ICU-acquired infection before pneumonia | 21 (33%) | 14 (29%) |  | 0.64 |
| Days after admission | 12 [7-23] | 13 [8-23] |  | 0.94 |
| Duration of MV before ICU acquired pneumonia | 11 [7-21] | 12 [7-20] |  | 0.70 |
| VAP | 54 (86%) | 44 (92%) |  | 0.33 |
| ***Clinical characteristics upon ICU admission*** | | | | |
| Main reason for ICU admission |  |  |  |  |
| Acute respiratory failure | 34 (63%) | 19 (40%) |  | 0.13 |
| Neurologic disorder/ coma | 3 (5%) | 5 (10%) |  | 0.26 |
| Cardiac arrest | 3 (5%) | 2 (4%) |  | 0.88 |
| Severe sepsis / septic shock | 20 (32%) | 23 (48%) | 1.98 (0.90 – 4.36) | 0.085 |
| Gastrointestinal bleeding | 2 (3%) | 1 (2%) |  | 0.73 |
| Other shock | 5 (8%) | 3 (6%) |  | 0.73 |
| Others | 8 (13%) | 2 (4%) |  | 0.14 |
| Diffuse dermatitis | 4 (6%) | 4 (8%) |  | 0.69 |
| Shock | 24 (38%) | 28 (58%) | 2.28 (1.04 – 4.99) | 0.035* |
| ARDS | 31 (49%) | 25 (52%) |  | 0.76 |
| Infection at ICU admission | 42 (67%) | 35 (73%) |  | 0.48 |
| SAPS II, median [IQR] | 40 [31-53] | 49 [36-68] |  | 0.059 |
| SAPS II >43 (median SAPS II) | 25 (40%) | 29 (60%) | 2.32 (1.05 – 5.10) | 0.028* |
| ***Prior hospital admission*** |  |  |  |  |
| Within previous year | 30 (48%) | 21 (44%) |  | 0.69 |
| Within 3 months | 25 (40%) | 21 (44%) |  | 0.67 |
| In another country | 2 (3%) | 4 (8%) |  | 0.25 |
| Prior ICU stay | 13 (21%) | 10 (21%) |  | 0.98 |
| Prior Surgery (< 1yr) | 12 (19%) | 9 (19%) |  | 0.97 |
| Admission category |  |  |  | 0.23 |
| Home | 27 (43%) | 13 (27%) |  |  |
| HCA-RF | 11 (17%) | 10 (21%) |  |  |
| Hospital | 25 (40%) | 25 (52%) |  |  |
| ***Procedures before ICU admission*** | | | | |
| Urinary catheter >24h | 11 (17%) | 11 (23%) |  | 0.48 |
| Venous catheter >24h | 8 (13%) | 6 (13%) |  | 0.98 |
| Endotracheal tube | 5 (8%) | 3 (6%) |  | 0.73 |
| ***Comorbidities*** |  |  |  |  |
| Charlson Comorbidity Index | 2 (1-3) | 2 (1-4) |  | 0.84 |
| Chronic pulmonary disease | 14 (2%) | 8 (17%) |  | 0.47 |
| Diabetes mellitus | 8 (13%) | 13 (27%) | 2.55 (0.94 – 6.9) | 0.055* |
| Neurologic disease | 13 (21%) | 10 (21%) |  | 0.98 |
| Immunodeficiency | 19 (30%) | 17 (35%) |  | 0.56 |
| Liver cirrhosis | 6 (10%) | 3 (6%) |  | 0.53 |
| Chronic renal insufficiency | 11 (17%) | 10 (21%) |  | 0.65 |
| Dialysis | 5 (8%) | 5 (10%) |  | 0.65 |
| Congestive heart failure | 15 (24%) | 17 (35%) |  | 0.18 |
| Urinary tract disease | 4 (6%) | 3 (6%) |  | 0.98 |
| ***Antibiotics before ICU*** |  |  |  |  |
| Ab <1 yr and broad-spectrum | 29 (46%) | 31 (65%) | 2.1 (0.99 - 4.6) | 0.052* |
| Ab < 3 mo. | 29 (46%) | 30 (63%) | 1.95 (0.91 -4.2) | 0.085 |
| Ab < 3 mo. and broad-sp. | 27 (43%) | 29 (60%) | 2.03 (0.95 -4.37) | 0.067* |
| Ab < 3 mo., broad-sp.and >10 d | 18 (29%) | 19 (40%) |  | 0.22 |
| Aminopenicillins | 6 (10%) | 8 (17%) |  | 0.26 |
| Penicilline + iBL | 18 (29%) | 16 (33%) |  | 0.59 |
| Fluoroquinolones | 7 (11%) | 11 (23%) | 2.4 (0.85 – 6.7) | 0.095 |
| Fluoroquinolones >2 days | 3 (5%) | 10 (21%) | 5.3 (1.4 – 20.4) | 0.06* |
| 3GC | 12 (19%) | 14 (29%) |  | 0.21 |
| Carbapenem | 4 (6%) | 3 (6%) |  | 0.98 |
| Aminoglycoside | 3 (5%) | 5 (10%) |  | 0.26 |
| Others | 18 (29%) | 11 (23%) |  | 0.50 |
| 3 mo. <Ab < 1 yr | 8 (13%) | 7 (15%) |  | 0.77 |
| 3 mo. <Ab and broad-sp < 1 yr | 7 (11%) | 7 (15%) |  | 0.59 |
| ***ESBL colonization*** |  |  |  |  |
| Known before admission | 4 (6%) | 7 (15%) |  | 0.16 |
| ESBL infection prior to ICU admission | 1 (2%) | 4 (8%) |  | 0.13 |
| Others ESBL infections before ICUAP | 3 (5%) | 7 (15%) | 3.4 (0.83 – 14.0) | 0.07 |
| **At admission** | 31 (49%) | 23 (48%) |  | 0.90 |
| *E. coli* alone  *E.cloacae/ K. pneumoniae* | 19 (30%)  12 (19%) | 3 (6%)  20 (42%) |  |  |
| **Acquisition** | 32 (51%) | 25 (52%) |  | >0.99 |
| *E. coli* alone  *E.cloacae/ K. pneumoniae* | 7 (11%)  25 (40%) | 0  25 (52%) |  |  |
| **Overall** |  |  |  |  |
| ***E. coli* alone** | 26 (41%) | 3 (6%) | 0.10 (0.03 – 0.34) | <0.001 |
| ***E.cloacae/ K. pneumoniae*** | 37 (59%) | 45 (78%) | 10.5 (2.95 - 37.6) | <0.001* |
| Days between ESBL colonization and ICUAP | 7 [3-12] | 7 [3-15] |  | 0.91 |
| ***Organ support before pneumonia*** | | | | |
| Mechanical ventilation | 55 (87%) | 46 (96%) |  | 0.12 |
| Dialysis | 13 (21%) | 19 (40%) | 2.5 (1.09-5.8) | 0.029* |
| ECMO | 0 (0%) | 4 (8%) |  | 0.032 |
| ARDS | 37 (59%) | 31 (65%) |  | 0.53 |
| ***Ab in ICU before pneumonia*** |  |  |  |  |
| Duration of therapy, days | 8 [6-15] | 10 [7-16] |  | 0.14 |
| Aminopenicillins | 15 (24%) | 10 (21%) |  | 0.71 |
| Penicillin + iBL | 30 (48%) | 9 (19%) | 0.25 (0.10-0.64) | 0.002 |
| Penicillin + iBL >2j | 23 (37%) | 6 (12%) | 0.25 (0.10–0.68) | 0.003* |
| Delay penicillin + iBL initiation – ICUAP, days | 13 [8-23] | 15 [8-24] |  | 0.85 |
| Fluoroquinolones | 7 (11%) | 4 (8%) |  | 0.63 |
| 3GC | 24 (38%) | 27(56%) | 2.10 (0.97–4.50) | 0.06 |
| Ureidopenicillin +iBL | 23 (37%) | 24 (50%) |  | 0.15 |
| Carbapenem | 16 (25%) | 19 (40%) |  | 0.11 |
| Aminoglycoside | 26 (41%) | 24 (50%) |  | 0.36 |
| Co-trimoxazole | 7 (11%) | 9 (19%) |  | 0.26 |
| Nitroimidazole | 4 (6%) | 6 (13%) |  | 0.27 |
| Glycopeptides | 8 (13%) | 11 (21%) |  | 0.14 |
| Macrolides | 17 (27%) | 11 (21%) |  | 0.63 |
| ***Ab in ICU before pneumonia and after colonization*** | | | | |
| Duration of therapy, days | 8 [6-15] | 11 [7-16] | 1.04 (0.99-1.08) | 0.08 |
| Aminopenicillins | 12 (19%) | 7 (15%) | 0.73 (0.26-2.01) | 0.54 |
| Penicillin + iBL | 21 (33%) | 7 (15%) | 0.34 (0.13-0.89) | 0.03 |
| Penicillin + iBL >2j | 11(18%) | 3 (6%) | 0.32 (0.08-1.2) | 0.09 |
| Fluoroquinolones | 3 (1%) | 4 (8%) | 1.82 (0.39-8.54) | 0.45 |
| 3GC | 13 (21%) | 20 (42%) | 2.75 (1.19-6.35) | 0.02* |
| Ureidopenicillin +iBL | 12 (19%) | 13 (27%) | 1.58 (0.64-3.86) | 0.32 |
| Carbapenem | 10 (25%) | 17 (40%) | 2.90 (1.18-7.13) | 0.02 |
| Aminoglycoside | 16 (25%) | 18 (38%) | 1.76 (0.78-3.98) | 0.17 |
| Co-trimoxazole | 7 (11%) | 9 (19%) | 1.85 (0.63-5.38) | 0.26 |
| Nitroimidazole | 3 (5%) | 4 (8%) | 1.82 (0.39-8.54) | 0.45 |
| Glycopeptides | 7 (11%) | 7 (15%) | 1.36 (0.44-4.20) | 0.59 |
| Macrolides | 8 (13%) | 7 (15%) | 1.17 (0.39-3.50) | 0.77 |

Abbreviations (Table e1):

Ab=antibiotic; broad-sp.= broad-spectrum (other antibiotic than ampicillin alone); 3GC=third-generation cephalosporin; iBL=beta-lactamase inhibitor; yr=year; mo= month; VAP= ventilator associated pneumonia; ICU-AP= ICU acquired pneumonia; ECMO= extracorporal membrane oxygenation; ARDS= acute respiratory distress syndrome; [IQR]=interquartile range (25%-75%); *= variable entered in multivariable logistic regression.

**Table S2**. Sensitivity analysis, excluding 6 pts having colonization identified on the same day as pneumonia (Logistic regression analysis).

| Associated factors | **aOR** | **[95% Conf. Interval]** | **P** |
| --- | --- | --- | --- |
| SAPS2 *>* 43 | 3.22 | 1.25 - 8.26 | 0.015 |
| >2 days amoxicillin/clavulanic acid in ICU | 0.20 | 0.06 -0.66 | 0.008 |
| Colonization with *E.cloacae* or *K. pneumoniae* | 17.4 | 3.63 - 83.2 | <0.0001 |

**Table S3.** Microorganisms associated with ICU-acquired pneumonia among 111 patients with ESBL-PE colonization

| **Microorganisms** | **ESBL-**  **(n=63)** | **ESBL +**  **(n=48)** |
| --- | --- | --- |
| **Enterobacteriaceae alone** | 17 (27%) | 31 (65%) |
| ESBL *Enterobacter cloacae / E. aerogenes* | 0 (0%) | 13 (27%) |
| ESBL *Klebsiella pneumoniae* | 0 (0%) | 12 (25%) |
| ESBL *Escherichia coli* | 0 (0%) | 4 (8%) |
| Polymicrobial ESBL-PE | 0 (0%) | 2 (4%) |
| **Non-fermenting gram-negative bacilli and enterobacteriaceae** | 6 (10%) | 17 (35%) |
| Polymicrobial with ESBL *Enterobacter* or *K. pneumoniae* and non-fermenting gram-negative bacilli | 0 (0%) | 17 (35%) |
| Polymicrobial with ESBL *E. coli* and non-fermenting gram-negative bacilli | 0 (0%) | 0 (0%) |
| **NF-GNB alone** | 37 (59%) | 0 (0%) |
| **Gram positive bacteria** | 3 (5%) | 0 (0%) |
| **Carbapenem-resistant microorganism*** | 19 (30%) | 6 (13%) |
| **Carbapenem-resistant NF GNB** | 17 (89%) | 6 (100%) |

*****There were two carbapenem-R non-NF GNB microorganisms (1 *E.faecium* and 1 *Providencia rettgeri*)

NFGNB= Nonfermenting gram-negative bacilli

**Table S4.** Univariate analyses of variables associated pneumonia due to a carbapenem-resistant bacteria.

| **Variables** | **CRB-**  **(n=86)** | **CRB +**  **(n=25)** | **OR (95%CI)** | **P Value** |
| --- | --- | --- | --- | --- |
| Male gender | 62 (72%) | 19 (76%) |  | 0.69 |
| Age, median [IQR] | 64 [53-74] | 60 [42 - 74] |  | 0.34 |
| Days after admission, median | 12 [7 - 21] | 11.5 [8 - 25] |  | 0.45 |
| Duration of MV before ICU acquired pneumonia | 11 [6 - 20] | 11.5 [9 - 25] |  | 0.30 |
| VAP | 74 (86%) | 24 (96%) |  | 0.29 |
| ***Clinical characteristics upon ICU admission*** | | | | |
| Main reason for ICU admission |  |  |  |  |
| Acute respiratory failure | 40 (47%) | 13 (52%) |  | 0.63 |
| Neurologic disorder/ coma | 7 (8%) | 1 (4%) |  | 0.68 |
| Cardiac arrest | 3 (3%) | 2 (8%) |  | 0.31 |
| Severe sepsis / septic shock | 33 (38%) | 10 (40%) |  | 0.88 |
| Gastrointestinal bleeding | 2 (2%) | 1 (4%) |  | 0.54 |
| Other shock | 5 (6%) | 3 (12%) |  | 0.38 |
| Others | 9 (10%) | 1 (4%) |  | 0.45 |
| Diffuse dermatitis | 7 (8%) | 1 (4%) |  | 0.68 |
| Shock | 39 (45%) | 13 (52%) |  | 0.56 |
| ARDS | 37 (43%) | 19 (76%) | 4.2 (1.5-11.5) | **0.004** |
| Infection at ICU admission | 56 (65%) | 21 (84%) |  | 0.07 |
| SAPS II, median [IQR] | 42 [32 - 53] | 46.5 [ 35 - 78] |  | 0.34 |
| ***SAPS2 >43*** | 41 (48 %) | 13 (52 %) |  | 0.70 |
| ***Prior hospital admission*** |  |  |  |  |
| Within previous year | 37 (43%) | 14 (56%) |  | 0.25 |
| Within 3 months | 32 (37%) | 14 (56%) | 2.2 (0.87 – 5.3) | 0.09 |
| In another country | 5 (6%) | 1 (4%) |  | 1.0 |
| Prior ICU stay | 15 (17%) | 8 (32%) |  | 1.0 |
| Prior Surgery (< 1yr) | 13 (15%) | 8 (32%) | 2.6 (0.95 – 7.4) | 0.08 |
| Admission category |  |  |  | 0.37 |
| Home | 33 (38%) | 7 (28%) |  |  |
| HCA-RF | 14 (16%) | 7 (28%) |  |  |
| Hospital | 39 (45%) | 11 (44%) |  |  |
| ***Procedures before ICU admission*** | | | | |
| Urinary catheter >24h | 15 (17%) | 7 (13%) |  | 0.26 |
| Venous catheter >24h | 9 (10%) | 5 (20%) |  | 0.30 |
| Endotracheal tube | 7 (8%) | 1 (4%) |  | 0.68 |
| ***Comorbidities*** |  |  |  |  |
| Charlson Comorbidity Index | 2 (1 - 3) | 3 (1 – 3.5) |  | 0.31 |
| Charlson >2 | 30 (35%) | 14 (56%) | 2.4 ( 0.96 – 5.9) | 0.057 |
| Chronic pulmonary disease | 16 (19%) | 6 (24%) |  | 0.57 |
| Diabetes mellitus | 18 (21%) | 3 (12%) |  | 0.40 |
| Neurologic disease | 20 (23%) | 3 (12%) |  | 0.22 |
| Immunodeficiency | 26 (30%) | 10 (40%) |  | 0.36 |
| Liver cirrhosis | 6 (7%) | 3 (12%) |  | 0.42 |
| Chronic renal insufficiency | 11 (13%) | 10 (40%) | 4.5 (1.6-12.6) | **0.002** |
| Dialysis | 5 (6%) | 5 (20%) | 4.05 (1.07-15.3) | 0.044 |
| Congestive heart failure | 26 (30%) | 6 (24%) |  | 0.55 |
| Urinary tract disease | 3 (3%) | 4 (16%) | 5.3 (1.1-25.4) | 0.044 |
| ***Antibiotics before ICU*** |  |  |  |  |
| Antibiotic therapy within 1 yr | 45 (52%) | 17 (68%) |  | 0.17 |
| Ab <1 yr and broad-spectrum | 44 (51%) | 16 (64%) |  | 0.26 |
| Ab < 3 mo. | 42 (49%) | 17 (68%) | 2.2 (0.87 – 5.7 ) | 0.09 |
| Ab < 3 mo. and broad-sp. | 40 (47%) | 16 (64%) |  | 0.12 |
| Ab < 3 mo., broad-sp.and >10 d | 25 (29%) | 12 (48%) | 2.25 ( 0.91 – 5.6) | 0.08 |
| Aminopenicillins | 9 (10%) | 5 (20%) |  | 0.30 |
| Penicilline + iBL | 26 (30%) | 8 (32%) |  | 0.87 |
| Fluoroquinolones | 13 (15%) | 5 (20%) |  | 0.55 |
| 3GC | 14 (16%) | 12 (48%) | 4.7 (1.8-12.5) | **0.001** |
| Carbapenem | 1 (1%) | 6 (24%) | 26.8 (3.1-236,2) | **<0.001** |
| Aminoglycoside | 4 (5%) | 4 (16%) |  | 0.20 |
| Others | 20 (23%) | 9 (36%) |  | 0.49 |
| 3 mo. <Ab < 1 yr | 10 (12%) | 5 (20%) |  | 0.32 |
| 3 mo. <Ab and broad-sp < 1 yr | 10 (12%) | 4 (16%) |  | 0.51 |
| ***ESBL colonization*** |  |  |  |  |
| Known before admission | 7 (8%) | 4 (16%) |  | 0.26 |
| ESBL infection prior to ICU admission | 2 (2%) | 3 (12%) |  | 0.07 |
| Others ESBL infections before ICUAP | 5 (6%) | 5 (20%) | 4.1 (1.1-15.4) | 0.044 |
| At admission | 42 (49%) | 12 (48%) |  | 0.94 |
| E. coli alone | 21 (24%) | 8 (32%) |  | 0.45 |
| E.cloacae/ K. pneumoniae | 65 (76%) | 17 (68%) |  | 0.45 |
| Days between ESBL colonization and ICUAP | 7 [3 - 12] | 8 (4 - 18] |  | 0.19 |
| ***Organ support before pneumonia*** | | | | |
| ICU-acquired infection before pneumonia | 23 (27%) | 12 (48%) | 2.5 (1.0-6.3) | 0.044 |
| ICU-acquired pneumonia before >1 | 9 (10%) | 4 (16%) |  | 0.48 |
| Mechanical ventilation | 80 (93%) | 25 (100%) |  | 0.33 |
| Dialysis | 18 (21%) | 14 (56%) | 4.8 (1.9-12.4) | **0.001** |
| ECMO | 3 (3%) | 1 (5%) |  | 1.0 |
| ARDS | 44 (51%) | 24 (96%) | 22.9 (3.0-177.0) | **<0.001** |
| ***Ab in ICU before pneumonia*** | | | | |
| Days of therapy | 8 [6-15] | 11 [7-16] |  | 0.019 |
| Aminopenicillins | 22 (26%) | 3 (12%) |  | 0.15 |
| Penicillin + iBL | 22 (26%) | 3 (12%) | 0.25 (0.10-0.64) | 0.92 |
| Fluoroquinolones | 4 (5%) | 7 (28%) | 8.0 (2.1-30.2) | **0.002** |
| 3GC | 37 (43%) | 14(56%) |  | 0.25 |
| Ureidopenicillin +iBL | 35 (41%) | 12 (48%) |  | 0.51 |
| Carbapenem | 19 (22%) | 16 (64%) | 6.3 (2.4-16.4) | **<0.001** |
| Aminoglycoside | 35 (41%) | 15 (60%) |  | 0.09 |
| Co-trimoxazole | 12 (14%) | 4 (16%) |  | 0.76 |
| Nitroimidazole | 6 (7%) | 4 (16%) |  | 0.13 |
| Glycopeptides | 12 (14%) | 7 (28%) |  | 0.87 |
| Macrolides | 22 (26%) | 6 (24%) |  | 0.66 |
| Death in ICU | 37 (43.5%) | 15 (57.7%) |  | 0.20 |

Abbreviations: Ab=antibiotic; broad-sp.= broad-spectrum (other antibiotic than ampicillin alone); 3GC=third-generation cephalosporin; iBL=beta-lactamase inhibitor; 3 mo. <Ab < 1 yr = antibiotic within 3–12 months before ICU admission; yr=year; mo= month; VAP= ventilator associated pneumonia; ICU-AP= ICU acquired pneumonia; ECMO= extracorporal membrane oxygenation; ARDS= acute respiratory distress syndrome [IQR]=interquartile range (25%-75%).

**Table S5.** Antibiotic consumption in number of DDD / 1000 PD for most frequently used antibiotic classes (in our ICU between 2009 and 2014 and in ATB-Raisin surveillance network in 2014.

|  | **In our ICU** | | | | | | **French ICU** | |
| --- | --- | --- | --- | --- | --- | --- | --- | --- |
| **Year** | **2009** | **2010** | **2011** | **2012** | **2013** | **2014** | **UH**  **2014**  (1) | **NUH**  **2014**  (1) |
| **Amoxicillin** | 288 | 179 | 214 | 213 | 278 | 201 | 206 [160-234] | 181 [121-253] |
| **Amoxicillin enzyme inhibitor** | 243 | 196 | 174 | 219 | 224 | 203 | 177 [164-240] | 229 [175-287] |
| **Ureidopenicillin +iBL** | 78 | 72 | 83 | 96 | 90 | 98 | 82 [89-110] | 112 [81-137] |
| **Cefotaxime/**  **ceftriaxone** | 80 | 62 | 89 | 83 | 124 | 99 | 116 [44-194] | 167 [63-207] |
| **antipseudomonal 3GC** | 28 | 33 | 48 | 44 | 27 | 53 | 49 [29-115] | 42 [22-66] |
| **Carbapenems** | 65 | 77 | 85 | 77 | 76 | 73 | 98 [50-125] | 56 [31-90] |
| **Co-trimoxazole** | 50 | 46 | 67 | 82 | 84 | 36 | 49 [31-60] | 21 [9-38] |
| **Macrolides** | 66 | 59 | 108 | 97 | 125 | 76 | 73 [40-86] | 73 [40-110] |
| **Aminoglycoside** | 118 | 61.5 | 75 | 100 | 76 | 60 | 101 [58-161] | 107 [74-146] |
| **Fluoroquinolones** | 64 | 81 | 69 | 60 | 56 | 83 | 84 [61-127] | 166 [89-242] |
| **Glycopeptides** | 51 | 38 | 76 | 47 | 51 | 35 | 52 [23-74] | 40 [21-63] |
| **Nitroimidazole** | 20 | 21.5 | 26 | 25 | 35 | 31 | 42 [30-70] | 68 [44-100] |
| **ALL ANTIBIOTICS** | 1361 | 1088 | 1388 | 1353 | 1490 | 1212 | 1535 [1256-1742] | 1534 [1304-1903] |

Abbreviations: iBL=beta-lactamase inhibitor; UH university hospital, NUH non university hospital

1. Surveillance de la consommation des antibiotiques - Réseau ATB-Raisin / 2016 / Maladies infectieuses / Rapports et synthèses / Publications et outils / Accueil [Internet]. [cité 20 mars 2017]. Disponible sur: http://invs.santepubliquefrance.fr/Publications-et-outils/Rapports-et-syntheses/Maladies-infectieuses/2016/Surveillance-de-la-consommation-des-antibiotiques-Reseau-ATB-Raisin

**Table S6.** Study Population Characteristics

| **Variables** | Population n= 6303 |
| --- | --- |
| Age | 61 [41-74] |
| Male gender | 3750 (60%) |
| SAPS II, | 35 [22-52] |
| ***Main admission diagnosis in ICU*** |  |
| Acute respiratory failure | 1788 (28%) |
| Acute on chronic respiratory failure | 234 (4%) |
| Acute renal failure | 820 (13%) |
| Coma | 681 (11%) |
| Severe sepsis | 461 (7%) |
| Septic shock | 627 (10%) |
| Acute respiratory distress syndrome | 978 (16%) |
| Cardiac arrest | 511 (8%) |
| Cardiogenic shock | 485 (8%) |
| Other shock | 727 (12%) |
| Acute cardiac failure | 421 (7%) |
| Gastrointestinal bleeding | 148 (2%) |
| Pulmonary infection | 1824 (29%) |
| ***Comorbidities*** |  |
| Diabetes | 330 (5%) |
| Cirrhosis | 175 (3%) |
| ***Organ support*** |  |
| Mechanical ventilation | 2364 (38%) |
| Noninvasive ventilation | 1224 (19%) |
| Vasopressor use | 1747 (28%) |
| Renal replacement therapy | 449 (7%) |
| **Death in ICU** | 1172 (19%) |

Definition of abbreviations: ICU = intensive care unit; SAPS II = Simplified Acute

Physiology Score II; Data are presented as n (%) median (IQR).
